# The Influence of Liquid Medium Choice in Determination of Minimum Inhibitory Concentration of Essential Oils against Pathogenic Bacteria

**DOI:** 10.3390/antibiotics11020150

**Published:** 2022-01-25

**Authors:** Radka Hulankova

**Affiliations:** Department of Animal Origin Food and Gastronomic Sciences, Faculty of Veterinary Hygiene and Ecology, University of Veterinary Sciences Brno, 61242 Brno, Czech Republic; hulankovar@vfu.cz; Tel.: +420-541-562-750

**Keywords:** minimum inhibitory concentration (MIC), broth microdilution method, essential oil, antimicrobial activity, growth kinetics

## Abstract

So far there is no internationally accepted, standardized method for MIC determination of natural substances such as essential oils (EOs). The aim of this study was to elucidate how much the MIC values obtained from various studies using different culture media are comparable. The median MICs for cinnamon essential oil (EO) obtained by broth dilution were 517, 465 and 517 µg/mL for Mueller–Hinton Broth (MHB), Tryptone Soya Broth (TSB) and Brain Heart Infusion (BHI), respectively. The MIC values for oregano EO were significantly (*p* < 0.001) lower in MHB than in highly nutritious media; the median MICs were 616 µg/mL for MHB and 474 µg/mL for TSB and BHI. This statistically significant difference was noted for all the pathogens studied (*Salmonella* Enteritidis, *Escherichia coli* O157, *Listeria monocytogenes*, *Staphylococcus aureus*). In the presence of oregano EO lag phase was also much less prolonged in MHB (by 6–17%) than in the other media (by 92–189%). Some components of EOs may bind to starch in MHB; since the phenomenon seems to be selective and EO dependent, the use of MHB for comparison of antimicrobial properties of various EOs thus cannot be recommended.

## 1. Introduction

The antimicrobial effect of essential oils (EOs) in vitro has been thoroughly studied in the past decades. The most common methods used for research of antimicrobial activity of EOs include diffusion methods (agar disk-diffusion method, antimicrobial gradient method (Etest), agar well diffusion method) and dilution techniques (agar dilution and broth macro and microdilution method). Whereas the diffusion methods are mostly based on measurement of an inhibition zone on agar plate, dilution methods are based on determination of MIC via growth/no growth end-point [1]. Agar and broth dilution are the most commonly used methods for determination of MIC of antimicrobial agents. Broth dilution uses liquid culture medium containing increasing concentrations (typically a twofold dilution series) of the antimicrobial substance and inoculation with a defined number of bacterial cells. The method is termed macrodilution when using a total volume in milliliters, or microdilution, if performed in microtiter plates using ≤500 µL per well. The presence of turbidity or a sediment after incubation indicates growth of the microorganism. In both the agar and the broth dilution techniques, the MIC is defined as the lowest concentration of the antimicrobial substance that prevents visible growth of a microorganism under defined conditions [2].

The methods used for research of EOs are derived from the methods used for testing of antimicrobial susceptibility of microorganisms against antibiotics. These procedures have been honed for many years and standardized to allow for reproducibility. The Clinical and Laboratory Standards Institute’s (CLSI’s) protocols are accepted world-wide for testing of antibiotics. Both CLSI and EUCAST performance standards include cation-adjusted Mueller–Hinton broth and Mueller–Hinton agar as the appropriate growth medium for broth and agar dilution technique, respectively [3,4]. Although many studies on EOs uses these media, other broths and agars are commonly employed. Indeed, it is often very difficult to compare the results of antimicrobial effect in published articles due to the use of different non-standardized procedures [1]. Van de Vel et al. [5] created a review on methods for in vitro evaluating antimicrobial activity, based on analysis of data published between 1995–2016. The authors clearly identified the most important factors causing the variance in minimum inhibitory concentration (MIC) between studies, which included incubation conditions, culture media and the use of emulsifiers or solvents. Balouiri et al. [1] mention also other factors, e.g., inoculum size and end-points determination.

There have been numerous calls for an international standard method for evaluation of the antimicrobial activity of EOs and its compounds in order to achieve better comparability between studies [1,5]. One of the main parameters to establish would be the growth medium. Although Mueller–Hinton broth seems to be the logical choice for broth dilution, no evaluation was performed so far regarding the possible interactions between EOs components and the components in the culture medium, similar to interactions with food components (proteins, fat, starch) which have been previously reported [6]. 

The aim of this study was not a development of a new culture medium suitable for EOs evaluation, but a comparison of the most commonly used, clearly defined laboratory growth media including Mueller–Hinton broth in order to elucidate how much the MIC values obtained in different culture media are comparable.

## 2. Results and Discussion

This study focused on comparison of MHB, BHI and TSB as the most common media used for determination of MIC of EOs by broth dilution method. Generally, TSB showed significantly lower MIC values than MHB (*p* < 0.001) and BHI (*p* = 0.006). However, further analysis revealed that EO also played a role. The most notable are the increased MIC values for oregano EO in MHB (median 616 µg/mL) in comparison to other media with median 474 µg/mL (Table 1). 

The composition of BHI and TSB is similar, with BHI containing slightly more proteinous components (28 g/L) than TSB with 20 g/L (Table 2). MHB is on the other hand a far less complex medium containing a high amount of proteinous components (317.5 g/L) and starch (1.5 g/L). Proteins are known to interfere with the antimicrobial activity of EOs [6,7,8,9,10]. Increased MIC values of thymol and carvacrol in nutrient agar and TSB, respectively, were reported after addition of bovine serum albumin [7,8]. Oregano and thyme EOs were also less inhibitory against *L. monocytogenes* in TSB after addition of starch (1%, 5% and 10%), whereas the same oils did not bind to simple sugars represented mainly by glucose and fructose [6,9]. On the other hand, the increased MIC was not observed for cinnamon EO, suggesting that the compounds of this oil do not bind to starch or proteins in the same manner as the oregano EO. Carvacrol, the main component of oregano EO, is a phenolic monoterpenoid, whereas cinnamaldehyde, the main component of cinnamon EO, has an aldehyde group [5]. Furthermore, as there are differences between simple and complex saccharides, it should be further elucidated how the degree of hydrolyzation influences the binding of EO components to proteinous substances, as not only amount, but also the form could play a role in the interaction. While bovine serum albumin interfered negatively with EO compounds [7,8], addition of meat extract to TSB increased the efficacy of EOs [6] and of aqueous extract from rose fruits [10], although in the latter case the effect was clearly pronounced only for high concentration of the extract used. Although no deliberate comparison of the media in regard to EOs have been made so far, Serio et al. [11] reported growth of *L. monocytogenes* in both BHI and TSB after exposure to oregano EO. Lag phases in TSB were longer than in BHI, which corresponds with the results of our study (Table 3). The authors mention that BHI may favor the growth of cells stressed by EO due to the presence of osmoprotective carnitine, which can improve the cell resistance.

In a study by Granata et al. [12], encapsulated oregano (thymol chemotype) and thyme (carvacrol chemotype) EOs were tested on various pathogens. The MIC of *L. monocytogenes* determined in BHI was significantly lower (0.03 mg/mL) than the MICs of *S. aureus* and *E. coli* grown in MHB (0.06 and 0.12 mg/mL, respectively). On the other hand, in a study of Simionato et al. [13], encapsulated cinnamon EO exhibited the same MIC for *L. monocytogenes* in BHI as for *E. coli* O157 and *Y. enterocolitica* in MHB. Although the encapsulation, strain specificity and other factors could play a role in the differences (or the lack of them), these data could also corroborate the specific binding of EO compounds to MHB compounds. Our results from the MIC assay were supported by the values of growth kinetic parameters. While the lag phase duration doubled in BHI and almost tripled in TSB in the presence of oregano EO in comparison to control (Table 3), it was prolonged only by 6–17% in MHB. The maximum growth rate also decreased the less in MHB in the presence of oregano EO in comparison to control.

**Figure 1:**
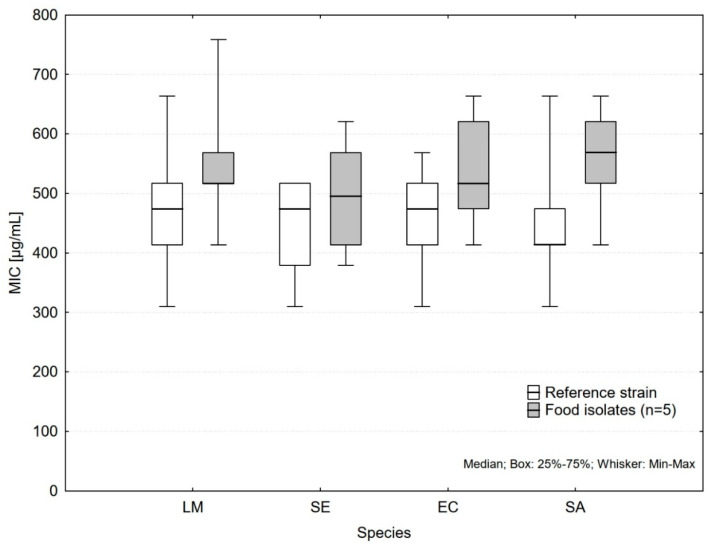
Differences in MIC between reference strains and mixtures of food isolates (LM, *Listeria monocytogenes*; SE, *Salmonella* Enteritidis; EC, *Escherichia coli* O157; SA, *Staphylococcus aureus*).

The differences between the reference strain and the mixture of food isolates (Figure 1) were statistically significant for all the species except *Salmonella* Enteritidis. The most pronounced difference was found for *S. aureus* (*p* < 0.001) with median 414 µg/mL for reference strain and 569 µg/mL for mixture of food isolates. These differences were not affected by the type of growth medium or essential oil. The use of mixture of contemporary food isolates represents a worst-case-scenario, where the most resistant strain will prevail. Since testing of numerous isolates individually is laborious, this approach may bring more precise results than the use of a single reference strain, although well defined.

**Figure 2:**
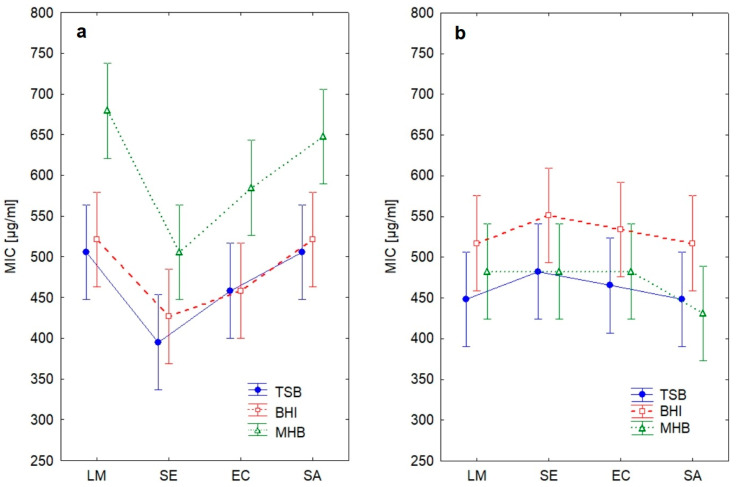
Differences in MIC between essential oils, growth media and pathogens (LM, *Listeria monocytogenes*; SE, *Salmonella* Enteritidis; EC, *Escherichia coli* O157; SA, *Staphylococcus aureus*). (**a**) Oregano essential oil; (**b**) cinnamon essential oil.

During evaluation of the interaction of medium type with other factors, statistically significant interaction was found between species and EO (Table 4), but not between species, EO and medium (Figure 2), meaning that the differences between species were approx. the same in all the media. It should be mentioned that *L. monocytogenes* is usually cultivated in TSB with yeast extract; however, the growth in TSB in our study was luxuriant enough to enable the species comparison using the same medium. On the other hand, no strains were able to grow in MHB without lysed blood supplementation. Although the supplementation is in CLSI standards for antimicrobial susceptibility testing [14], many studies testing EOs against *L. monocytogenes* in MHB do not mention this M45 standard or any supplementation [15,16,17,18,19,20] and in another study only MHB for *Streptococcus* spp. was supplemented, although *L. monocytogenes* was included in the study [21]. This ambiguity in medium specification or supplementation decreases between-studies comparability of results obtained in MHB.

Statistically significant interaction was found between species and EO (Table 4); whereas the differences between species were not significant when cinnamon EO was used, for oregano EO the MIC of *Salmonella* Enteritidis was significantly lower than the MICs of *L. monocytogenes* and *S. aureus* (*p* = 0.009 and 0.015, respectively). For the Gram positive bacteria, the MIC values were higher for oregano EO than for cinnamon EO (*p* = 0.002 and 0.003 for *L. monocytogenes* and *S. aureus*, respectively). On the other hand, the Gram negative bacteria showed opposite results (although the difference was not statistically significant). This is in accordance with the reported correlation of EOs’ composition with their antimicrobial properties published by Bagheri et al. [22], where phenols were the most inhibitory against *Salmonella enterica* and *E. coli*, whereas aldehydes were the most inhibitory against *S. aureus*.

## 3. Materials and Methods

### 3.1. Bacterial Strains

Reference strains of four major food pathogens were used for the media testing: *Escherichia coli* O157 (ATCC 700728), *Salmonella* Enteritidis (ATCC 13076), *Listeria monocytogenes* (ATCC 13932) and *Staphylococcus aureus* (ATCC 25923). Simultaneously, for each species a mixture of five wild strains isolated from meat and meat products and preparations was used in order to count for strain variability. Specification of the bacterial isolates is available in Appendix A, Table A1. The bacterial cultures were kept frozen at −70 °C. 

### 3.2. Essential Oils

**Figure A1:**
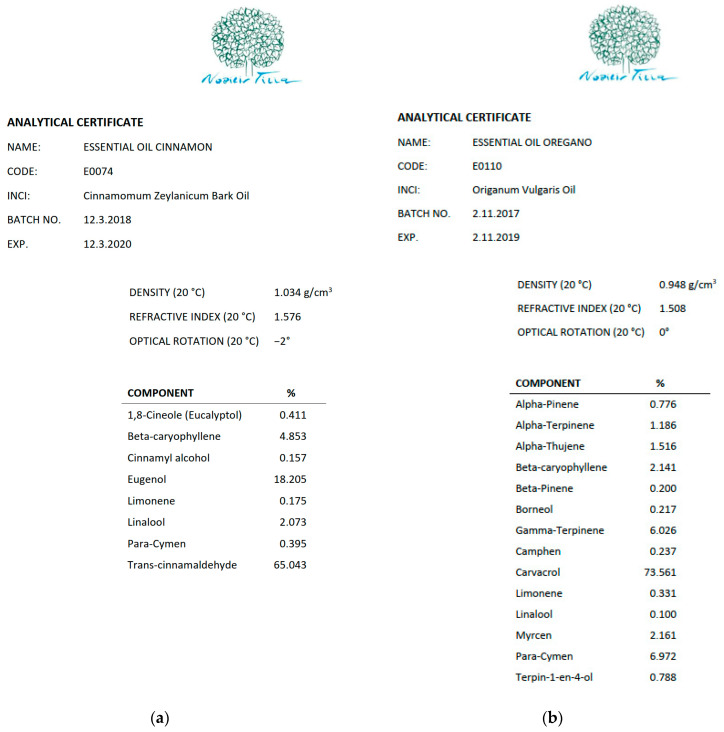
Analytical certificates. (**a**) chemical composition of cinnamon essential oil; (**b**) chemical composition of oregano essential oil.

Commercial essential oils (EOs) from oregano (*Origanum vulgare*, Spain) and cinnamon (*Cinnamomum zeylanicum*, Sri Lanka) obtained by steam distillation were purchased from Nobilis Tilia, Krásná Lípa, Czech Republic. Complete chemical composition of each oil (determined by GC-MS in an accredited laboratory in Germany, where the oils were manufactured by Joh. Vögele KG) is available in Appendix A, Figure A1. In short, the main components of oregano EO (density 0.948 g/mL) were carvacrol (73.6%), *p*-cymene (7.0%) and *γ*-terpinene (6.0%). The main components of cinnamon EO (density 1.034 g/mL) were trans-cinnamaldehyde (65%), eugenol (18.0%) and *β*-caryophyllene (4.9%). 

### 3.3. Determination of Minimum Inhibitory Concentration

Minimum inhibitory concentration (MIC) was determined by broth microdilution method. All the culture media used in this study were purchased from Oxoid, UK. The strains were plated on Tryptone Soya Agar (TSA, CM0131), Mueller–Hinton Agar (MHA, CM0337) and Brain Heart Infusion Agar (BHIA, CM1136) and once more subcultivated at 37 °C/24 h. Bacterial suspension was prepared in saline using the McFarland turbidity scale and further diluted to approximately 1 × 10^6^ cells/mL. According to the agar used for subcultivation, Tryptone Soya Broth (TSB, CM0129), Mueller–Hinton Broth (MHB, CM0405) or Brain Heart Infusion broth (BHI, CM1135) were used as the diluent. Since *L. monocytogenes* didn’t grow in pure MHB, Laked Horse Blood (SR0048) was added into MHB to make for final concentration 5% according to the international standards [4,14].

Essential oils were individually diluted in TSB, MHB and BHI (1:1) and vortexed vigorously. The emulsions were further diluted to a working concentration of 1%, from which a concentration row from 0.02–0.2% (*v*/*v*) was prepared. The dilutions of EOs were mixed 1:1 with the inoculum in a 96-well microplate. Both positive (0% EO) and negative (uninoculated solutions of EO) controls were included. The plates were incubated at 37 °C for 24 h. MIC was determined as the lowest concentration required to prevent visible growth. The whole experiment was twice replicated (*n* = 3). The media composition is showed in Table 2.

### 3.4. Growth Kinetics Measurements

The growth curves were constructed based on optical density (OD) measurement at 850 nm in Personal Bioreactor RTS-1 (Biosan, Riga, Latvia). Both control measures and measures in media with 0.03% (284 µg/mL) of oregano essential oil (approx. 50% of MIC) were performed. Since EOs are highly volatile substances, maximum volume (30 mL), sealed tubes and no tube rotation were used in order to prevent increased evaporation. The media were spiked with inoculum prepared as described above (with the same final concentration of approximately 5 × 10^5^ cells/mL) and incubated at 37 °C in the machine until the stationary phase was achieved. The OD was measured at 10 min intervals. For growth kinetics only reference strains of *E. coli* O157 and *L. monocytogenes* were used, since the factory calibration of the instrument is designed for specific microorganism size of 0.4–0.8 × 1–3 μm. Each measurement was performed three times.

### 3.5. Statistical Analysis

Since the dependent variable (MIC) was represented by interval, censored and non-normally distributed data, non-parametric statistical methods were used. Multiple comparisons were done by Wilcoxon (paired) test and the *p*-values were adjusted using Holm correction [23] with the *p* level of 0.05 set as statistically significant. Since there are no well-recognized non-parametric tests for multiple factors/mixed design, the interaction of media type with other factors (EO, Species, Origin) was assessed from the pairwise plots and verified by multiple comparisons. The species were compared using Kruskal–Wallis ANOVA and multiple comparisons of mean ranks. The computations were done in Statistica, v. 7.1 (StatSoft, Tulsa, OK, USA) and in Microsoft Excel 2016 (Microsoft, Redmond, WA, USA). 

Growth curves (OD values) were fitted in Microrisk Lab online predictor v1.2 [24] using primary growth model of Baranyi as the best fit [25] in order to compute maximum specific growth rate (μ_max_) and lag phase duration (λ).

## 4. Conclusions

The results show that the MIC values obtained by broth dilution using highly nutritious media such as TSB and BHI are fully comparable. On the other hand, the MIC values for oregano EO were significantly lower in MHB for all the pathogens, probably due to an interaction of its components with starch and reduced ability of the bacteria to repair the cell damage in a nutrient-deficient medium. Although there is still no internationally accepted, standardized method for MIC determination of natural substances such as EOs, MHB and MHA are the most commonly used media for broth and agar dilution, respectively. Admittedly, the MIC values for oregano EO in MHB were lower by 122–138 µg/mL than in TSB and BHI, which is not an enormous difference. However, the difference may not only hamper comparison of results from different studies using different media, but also the comparison of EOs conducted within a study, as not all the EOs are affected by starch in the same manner. Thus, the use of MHB for determination of antimicrobial properties of EOs is not ideal. The lowest MIC values were obtained in TSB which seems the most suitable candidate for reference medium in any future standard method for antimicrobial testing of EOs by broth dilution method. The medium in its basic composition better supports the growth of fastidious bacteria such as *Listeria monocytogenes*, without the need to specify whether cation adjustment/blood supplementation was used or not.

## Figures and Tables

**Table 1:** Differences in MIC (µg/mL) between various essential oils and growth media (*n* = 24).

Medium	Oregano Essential Oil			Cinnamon Essential Oil		
	Mean	Median	Min–Max	Mean	Median	Min–Max
TSB	466	474 ^Aa^	379–569	461	465 ^Aa^	310–620
BHI	482	474 ^Aa^	379–664	530	517 ^Ba^	414–620
MHB	604	616 ^Ba^	474–758	470	517 ^Ab^	310–620

MIC, minimum inhibitory concentration; TSB, tryptone soya broth; BHI, brain heart infusion; MHB, Mueller–Hinton broth; min, minimal value; max, maximal value; ^a,b^ mark statistically significant differences within a row; ^A,B^ mark statistically significant differences within a column.

**Table 2:** Comparison of the liquid media composition [g/L].

Broth	Proteinous Components		Glucose	Starch	Phosphate Buffer	NaCl
BHI	12.5	Brain infusion solids	2.0	-	2.5	5.0
	5.0	Beef heart infusion solids				
	10.5	Proteose peptone				
TSB	17.0	Pancreatic digest of casein	2.5	-	2.5	5.0
	3.0	Enzymatic digest of soya				
MHB	300.0	Beef infusion	-	1.5	-	-
	17.5	Casein hydrolysate				

**Table 3:** Comparison of growth model parameters for various media (mean ± SEM of three measurements).

Strain	Medium	OEO [µg/mL]	λ [h]	μ_max_ [OD units/h]	RMSE	Δ λ [%]	Δ μ_max_ [%]
*L. monocytogenes* ATCC 13932	TSB	0	3.47 ± 0.34	0.37 ± 0.03	0.04 ± 0.00	+189%	−70%
		284	10.04 ± 0.56	0.11 ± 0.02	0.04 ± 0.01		
	BHI	0	4.59 ± 0.31	0.30 ± 0.01	0.03 ± 0.00	+92%	−60%
		284	8.81 ± 0.50	0.12 ± 0.01	0.05 ± 0.01		
	MHB	0	5.15 ± 0.37	0.10 ± 0.01	0.02 ± 0.00	+17%	−30%
		284	6.01 ± 0.41	0.07 ± 0.01	0.03 ± 0.00		
*E. coli* O157 ATCC 700728	TSB	0	3.43 ± 0.36	0.27 ± 0.03	0.02 ± 0.01	+172%	−59%
		284	9.35 ± 0.49	0.11 ± 0.01	0.04 ± 0.01		
	BHI	0	3.88 ± 0.35	0.27 ± 0.04	0.02 ± 0.00	+113%	−41%
		284	8.72 ± 0.58	0.16 ± 0.02	0.05 ± 0.01		
	MHB	0	4.29 ± 0.41	0.19 ± 0.02	0.03 ± 0.01	+6%	−26%
		284	4.54 ± 0.53	0.14 ± 0.04	0.01 ± 0.01		

SEM standard error of the mean; OEO, oregano essential oil; λ, lag phase duration estimation; μ_max_, maximum specific growth rate estimation; RMSE, root-mean-square error (OD units); Δ increase/decrease of the parameter in OEO in comparison to control.

**Table 4:** Differences in MIC (µg/mL) between various essential oils and pathogens (*n* = 18).

Medium	Oregano Essential Oil			Cinnamon Essential Oil		
	Mean	Median	Min–Max	Mean	Median	Min–Max
*Salmonella* Enteritidis	442	474 ^Aa^	379–569	506	517 ^Aa^	310–620
*Escherichia coli* O157	500	474 ^ABa^	379–664	494	517 ^Aa^	310–620
*Listeria monocytogenes*	569	569 ^Ba^	474–758	483	517 ^Ab^	310–620
*Staphylococcus aureus*	558	569 ^Ba^	379–664	465	414 ^Ab^	310–620

MIC, minimum inhibitory concentration; min, minimal value; max, maximal value; ^a,b^ mark statistically significant differences within a row; ^A,B^ mark statistically significant differences within a column.

## Data Availability

All the data are available from the corresponding author upon reasonable request.

## Appendix A

**Table A1:** Bacterial isolates used in the study.

Species	Specification	Isolated	Source
*Escherichia coli*	serotype O157	2015	pork meat preparation
*Escherichia coli*	serotype O157	2017	wild boar carcass
*Escherichia coli*	serotype O157	2017	wild boar carcass
*Escherichia coli*	serotype O157	2018	sushi
*Escherichia coli*	serotype O157	2019	duck carcass
*Listeria monocytogenes*	serotype 1/2a	2014	chicken carcass
*Listeria monocytogenes*	serotype 1/2a	2014	chicken carcass
*Listeria monocytogenes*	serotype 1/2b	2018	cooked meat product
*Listeria monocytogenes*	serotype 1/2b	2018	cooked meat product
*Listeria monocytogenes*	serotype 1/2c	2015	pork meat preparation
*Salmonella Enteritidis*	phage type 1b	2015	poultry meat preparation
*Salmonella Enteritidis*	phage type 4	2015	poultry meat preparation
*Salmonella Enteritidis*	phage type 4b	2016	minced turkey meat
*Salmonella Enteritidis*	phage type 8	2014	chicken carcass
*Salmonella Enteritidis*	phage type 13	2014	chicken carcass
*Staphylococcus aureus*	-	2015	pork meat preparation
*Staphylococcus aureus*	-	2015	cooked meat product
*Staphylococcus aureus*	-	2019	duck carcass
*Staphylococcus aureus*	-	2019	duck carcass
*Staphylococcus aureus*	-	2018	cooked meat product
